# Five new species of the genera *Falcileptoneta* and *Longileptoneta* (Araneae, Leptonetidae) from South Korea

**DOI:** 10.3897/zookeys.1010.59915

**Published:** 2021-01-13

**Authors:** Tianqi Lan, Zhe Zhao, Seung Tae Kim, Jung Sun Yoo, Sue Yeon Lee, Shuqiang Li

**Affiliations:** 1 Institute of Zoology, Chinese Academy of Sciences, Beijing 100101, China Institute of Zoology, Chinese Academy of Sciences Beijing China; 2 Life and Environment Research Institute, Konkuk University, Seoul 05029, South Korea Konkuk University Seoul South Korea; 3 Biological and Genetic Resources Utilization Division, National Institute of Biological Resources, Incheon 22689, South Korea National Institute of Biological Resources Incheon South Korea; 4 College of Agricultural Life Science, Jeonbuk National University, Jeonju-si 54896, South Korea Jeonbuk National University Jeonju-si South Korea

**Keywords:** Biodiversity, litter-dwelling fauna, morphology, spider, taxonomy

## Abstract

Five new leptonetid species belonging to *Falcileptoneta* Komatsu, 1970 and *Longileptoneta* Seo, 2015 are newly described from South Korea: *F. dolsan***sp. nov.** (Jeollanam-do), *F. naejangsan***sp. nov.** (Jeollabuk-do), *L. buyongsan***sp. nov.** (Chungcheongbuk-do), *L. byeonsanbando***sp. nov.** (Jeollabuk-do) and *L. jirisan***sp. nov.** (Gyeongsangnam-do). All new species are found in leaf litter and described from both male and female specimens.

## Introduction

The spider family Leptonetidae Simon, 1890 includes 22 genera and 363 species from North America, the Mediterranean region and Asia (Li 2020; Wang et al. 2020; WSC 2020). Members of the family are tiny (1–3 mm) and typically have six eyes, with the anterior four eyes in a recurved row, and the posterior two contiguous; some species have only four or two eyes or are eyeless (Seo 2016b). Most species live in secluded environments, such as irregular sheet webs in leaf litter, caves, or mines (Xu et al. 2019). In South Korea, there are 47 described species in four genera: *Falcileptoneta* Komatsu, 1970; *Leptoneta* Simon, 1872; *Longileptoneta* Seo, 2015 and *Masirana* Kishida, 1942 (WSC 2020). *Falcileptoneta* is a species-rich genus in the family Leptonetidae with 59 described species. The genus was erected by Komatsu (1970), with *Leptoneta striata* Oi, 1952 as the type species. Species of *Falcileptoneta* are mainly distributed in South Korea and Japan (Irie and Ono 2007). Among these, 23 species are found in South Korea (WSC 2020). *Longileptoneta* is a small genus in the family Leptonetidae with only 11 described species; 5 species are distributed in South Korea (WSC 2020). *Longileptoneta* was established by Seo (2015a) for a new species, *L. songniensis* Seo, 2015, and can be easily recognized by the strong spines restricted to the male palpal femur, the prolaterodistal spur and the prolateral curvature of the palpal tarsus (Seo 2016b). The aim of this paper is to describe two new species of the genus *Falcileptoneta* and three new species of the genus *Longileptoneta* from South Korea.

## Materials and methods

All specimens were collected by hand from the litter layers of mixed forest in South Korea. Type material is deposited in the the National Institute of Biological Resources (**NIBR**) in Incheon, South Korea. All specimens were preserved in 75% ethanol and examined under a Leica M205C stereomicroscope. Images were captured with an Olympus C7070 wide zoom digital camera (7.1 megapixels) mounted on a Leica M205C stereomicroscope and assembled using Helicon Focus 3.10.3 image stacking software (Khmelik et al. 2006). All measurements are in millimeters (mm). The left male palps are illustrated. Internal genitalia of females were removed and treated in lactic acid before illustration. Leg measurements are shown as: Total length (femur, patella, tibia, metatarsus, tarsus). The distribution map was generated with ArcView GIS 3.2 (ESRI 2002). Adobe Photoshop CC (Adobe Systems Incorporated) was used for digital editing of photos and maps. Terminology and taxonomic descriptions follow Wang et al. (2017) and Xu et al. (2019).

## Taxonomy

### Family Leptonetidae Simon, 1890

#### Genus Falcileptoneta

Taxon classificationAnimaliaAraneaeLeptonetidae

Komatsu, 1970

A4B64533-6C52-58D3-80CB-50F747265DD4

Falcileptoneta Komatsu, 1970: 1

##### Type species.

*Leptoneta striata* Oi, 1952 from Japan.

##### Diagnosis.

**Figure 1.**
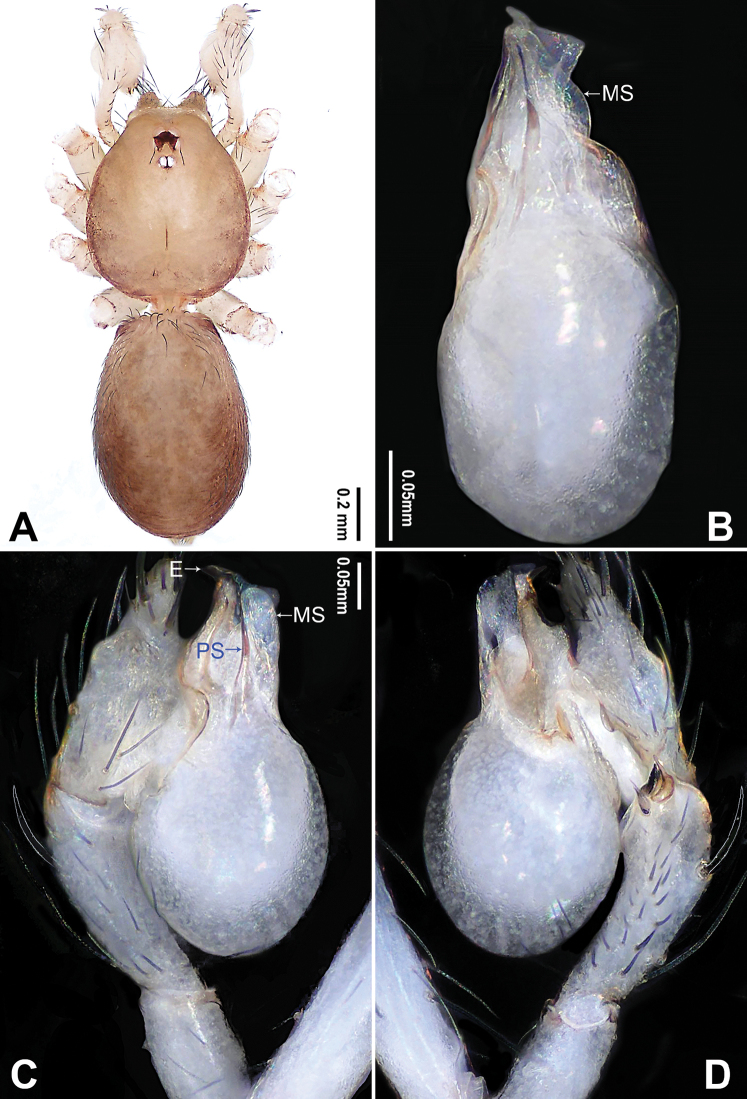
*Falcileptoneta dolsan* sp. nov., holotype male **A** habitus, dorsal view **B** palpal bulb, ventral view **C** palp, prolateral view **D** palp, retrolateral view. Abbreviations: **E** embolus **MS** median sclerite **PS** prolateral sclerite. Scale bars: equal for **C, D**.

The genus *Falcileptoneta* is similar to *Leptoneta* and *Longileptoneta* in having few sclerites on the male palpal bulb and absenting cribellum but can be distinguished by the following combination of male palpal characters: femur lacking strong spines, tibia usually with complex apophyses on the retrolateroapical end (Figs 1D, 3D), tarsus with shallow, transverse depression (Figs 1D, 3D) and the bulb usually with sickle-like or membranous embolus and complex laminae (Figs 1B–D, 3B–D).

#### Falcileptonetadolsan sp. nov.

Taxon classificationAnimaliaAraneaeLeptonetidae

2152E386-72CD-577B-89AD-9171EA80FB2D

http://zoobank.org/6C63FFC6-5990-40F9-8507-E718FE257F26

Figures 1 , 2 , 12

##### Type material.

***Holotype*.** Male (NIBR), South Korea, Jeollanam-do, Yeosu-si, Dolsan-eup, Seodeok-ri (34.641085°N, 127.760978°E, elevation ca 93 m), 13 August 2019, ZG. Chen, Z. Zhao & YY. Hu leg. ***Paratypes*.** 1 male and 1 female (NIBR), same data as holotype.

##### Diagnosis.

*Falcileptoneta dolsan* sp. nov. is similar to *F. digitalis* Seo, 2015 and *F. geumsanensis* Seo, 2016 but can be distinguished by the presence of three palpal tibial distal apophyses, dorsal apophysis sickle-shaped, middle apophysis black, triangular and ventral apophysis narrow and leaf-like (Fig. 1D) (vs. dorsal apophysis long and spur-like, middle apophysis black and rugulose, and ventral apophysis finger-like in *F. digitalis*; dorsal apophysis beak-like, middle apophysis leaf-like and ventral apophysis spur-like in *F. geumsanensis*); and by the bulb with a spine-like prolateral sclerite (Fig. 1C) (vs. narrow, leaf-like prolateral sclerite in *F. digitalis* and *F. geumsanensis*).

##### Description.

**Male** (holotype). Total length 1.67. Prosoma 0.75 long, 0.62 wide. Opisthosoma 0.92 long, 0.66 wide. Clypeus 0.10 high. Leg measurements: I 4.39 (1.21, 0.24, 1.20, 1.03, 0.71); II 3.42 (0.90, 0.22, 0.89, 0.77, 0.64); III 2.84 (0.83, 0.22, 0.71, 0.70, 0.38); IV 4.05 (1.15, 0.24, 1.09, 0.93, 0.64). Habitus as in Fig. 1A. Prosoma brown. Eyes six (Fig. 1A). Median groove, cervical grooves and radial furrows distinct. Opisthosoma brown, ovoid. Palp (Fig. 1C, D): femur without strong spine; tibia with three distal apophyses and one spine retrolaterally, dorsal apophysis sickle-shaped, middle apophysis black, triangular, ventral apophysis narrow, leaf-like (Fig. 1D), and with one strong dorsal spur (Fig. 1C, D); tarsus with transverse depression (Fig. 1D). Bulb with embolus bearing sickle-like tip and three types of sclerites: prolateral sclerite spine-like; median sclerite shoehorn-like; retrolateral sclerite transparent and membranous (Fig. 1B–D).

**Figure 2.**
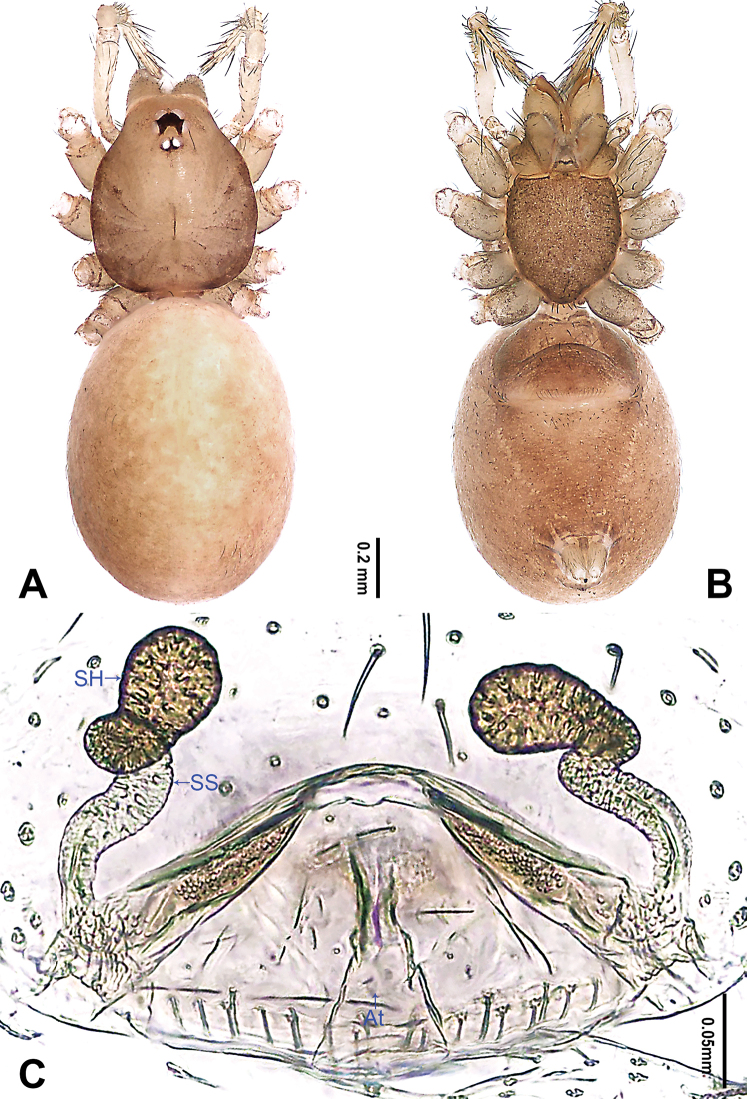
*Falcileptoneta dolsan* sp. nov., female paratype **A** habitus, dorsal view **B** habitus, ventral view **C** internal genitalia, dorsal view. Abbreviations: **At** atrium **SH** spermathecae **SS** spermathecae stalk. Scale bars: equal for **A, B**.

**Female** (paratype). Similar to male in color and general features, habitus as in Fig. 2A, B. Total length 2.09. Prosoma 0.74 long, 0.62 wide. Opisthosoma 1.35 long, 1.06 wide. Clypeus 0.12 high. Leg measurements: I 3.89 (1.05, 0.25, 1.03, 0.92, 0.64); II 3.24 (0.89, 0.24, 0.86, 0.67, 0.58); III 2.81 (0.79, 0.23, 0.69, 0.64, 0.46); IV 3.72 (1.03, 0.24, 1.01, 0.85, 0.59). Internal genitalia (Fig. 2C) with atrium rectangular, genital duct coiled apically, and spermathecae pear-shaped.

##### Etymology.

The specific name refers to the type locality and is a noun in apposition.

##### Habitat.

Litter layers in mixed forest.

##### Distribution.

South Korea (Jeollanam-do; Fig. 12).

#### Falcileptoneta naejangsan sp. nov.

Taxon classificationAnimaliaAraneaeLeptonetidae

D04D496F-AFD8-5C55-8CAC-C18DCC74AB4C

http://zoobank.org/A6EE1759-E916-42CC-9A0C-50D0E169169F

Figures 3 , 4 , 12

##### Type material.

***Holotype*.** Male (NIBR), South Korea, Jeollabuk-do, Jeongeup-si, Naejang-dong, Mt. Naejangsan National Park (35.491727°N, 126.900469°E, elevation ca 237 m), 9 August 2019, ZG. Chen, Z. Zhao & YY. Hu leg. ***Paratype*.** 1 female (NIBR), same data as holotype.

##### Diagnosis.

**Figure 3.**
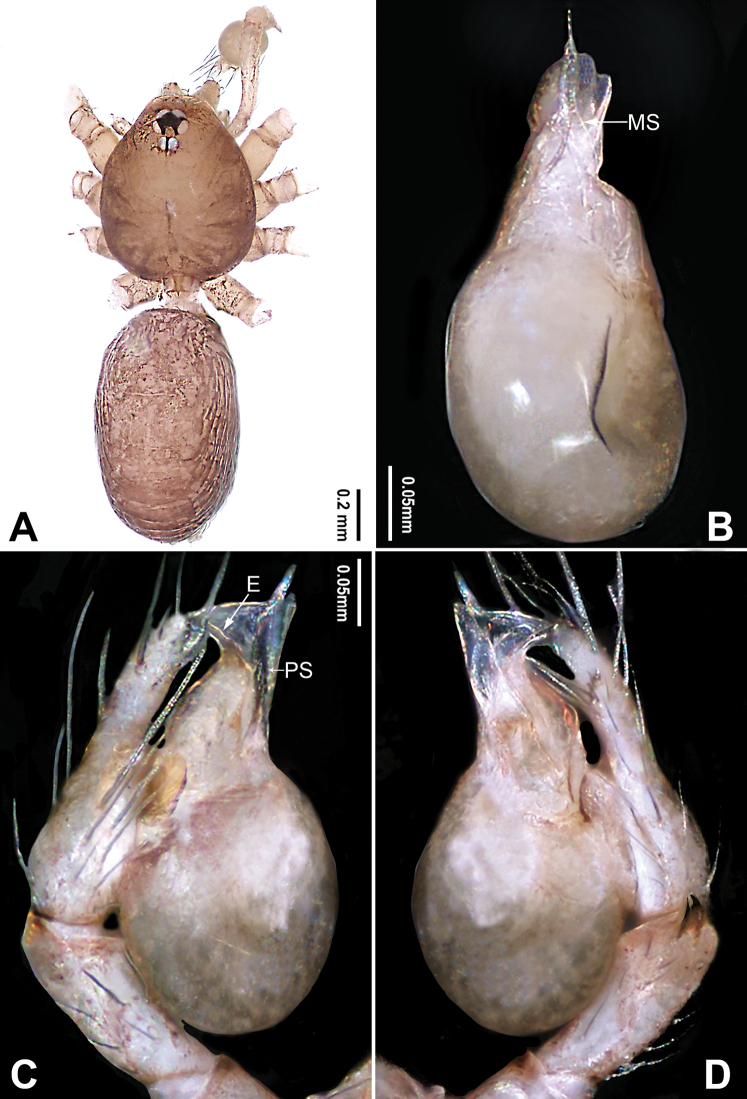
*Falcileptoneta naejangsan* sp. nov., holotype male **A** habitus, dorsal view **B** palpal bulb, ventral view **C** palp, prolateral view **D** palp, retrolateral view. Abbreviations: **E** embolus **MS** median sclerite **PS** prolateral sclerite. Scale bars: equal for **C, D**.

*Falcileptoneta naejangsan* sp. nov. is similar to *F. naejangensis* Seo, 2015 and *F. sunchangensis* Seo, 2016 but can be distinguished by the shape of the two palpal tibial retrolaterodistal apophyses, with the dorsal apophysis beak-like and the ventral apophysis spine-like (Fig. 3D) (vs. dorsal one curved and ventral one triangular, and with one spine in *F. naejangensis*; dorsal apophysis thick and spur-like, and ventral apophysis spine-like in *F. sunchangensis*); and by the male palpal bulb with a spine-like prolateral sclerite and narrow leaf-like median sclerite (Fig. 3B–D) (vs. needle-shaped prolateral sclerite and longish, shoehorn-like median sclerite in *F. naejangensis*; narrow, leaf-like prolateral sclerite and leaf-like median sclerite in *F. sunchangensis*).

##### Description.

**Male** (holotype). Total length 1.68. Prosoma 0.69 long, 0.58 wide. Opisthosoma 0.99 long, 0.58 wide. Clypeus 0.10 high. Leg measurements: I 4.43 (1.15, 0.32, 1.22, 1.03, 0.71); II 3.41 (0.90, 0.26, 0.90, 0.77, 0.58); III 2.76 (0.83, 0.26, 0.65, 0.64, 0.38); IV 3.97 (1.15, 0.26, 1.15, 0.83, 0.58). Habitus as in Fig. 3A. Prosoma dark brown. Eyes six (Fig. 3A). Median groove, cervical grooves and radial furrows distinct. Opisthosoma dark brown, ovoid. Palp (Fig. 3C, D): femur lacking strong spine; tibia with two retrolaterodistal apophyses, dorsal apophysis beak-like and ventral apophysis spine-like (Fig. 3D); tarsus with transverse depression (Fig. 3D). Bulb with embolus bearing sickle-like tip and three types of sclerites: prolateral sclerite spine-like; median sclerite narrow, leaf-like; retrolateral sclerite transparent and membranous (Fig. 3B–D).

**Figure 4.**
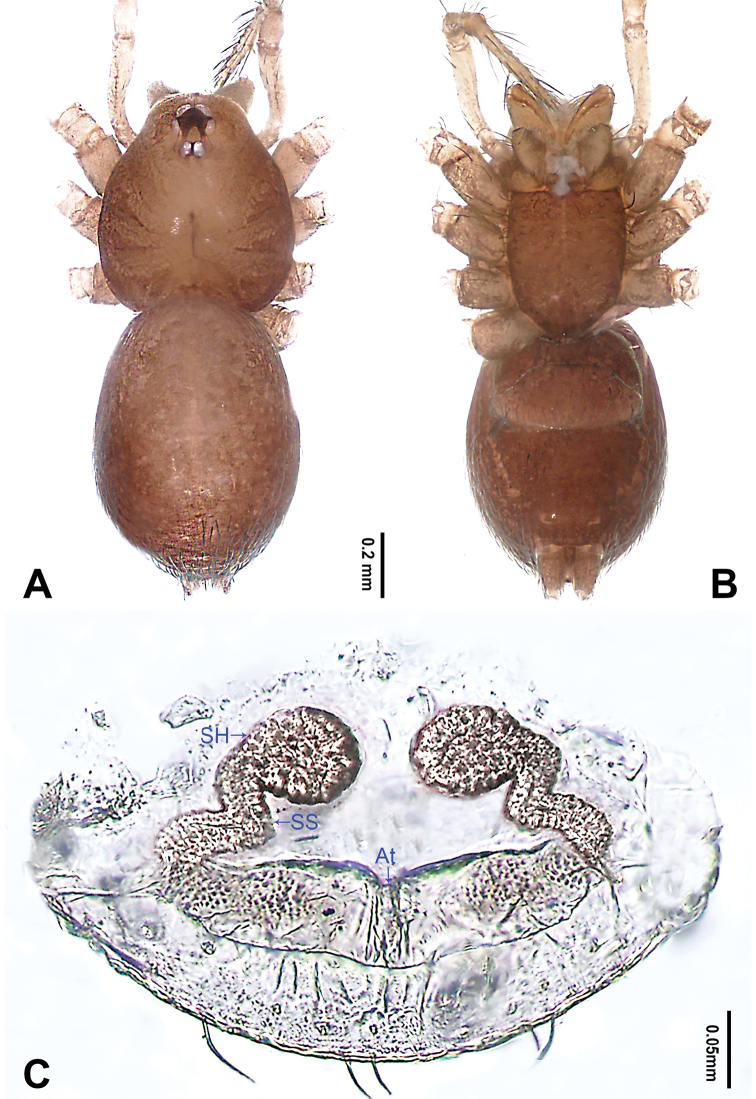
*Falcileptoneta naejangsan* sp. nov., female paratype **A** habitus, dorsal view **B** habitus, ventral view **C** internal genitalia, dorsal view. Abbreviations: **At** atrium **SH** spermathecae **SS** spermathecae stalk. Scale bars: equal for **A, B**.

**Female** (paratype). Similar to male in color and general features, habitus as in Fig. 4A, B. Total length 1.80. Prosoma 0.68 long, 0.61 wide. Opisthosoma 1.12 long, 0.63 wide. Clypeus 0.11 high. Leg measurements: I 4.16 (1.15, 0.38, 1.15, 0.90, 0.58); II 3.15 (0.90, 0.26, 0.83, 0.71, 0.45); III 2.68 (0.77, 0.19, 0.64, 0.63, 0.45); IV 3.78 (1.15, 0.26, 1.09, 0.77, 0.51). Internal genitalia (Fig. 4C) with atrium rectangular, genital duct coiled apically, and spermathecae round.

##### Etymology.

The specific name refers to the type locality and is a noun in apposition.

##### Habitat.

Litter layers in mixed forest.

##### Distribution.

South Korea (Jeollabuk-do; Fig. 12).

#### Genus Longileptoneta

Taxon classificationAnimaliaAraneaeLeptonetidae

Seo, 2015

2DE5C169-04A3-573B-9FAA-23E952982C7A

Longileptoneta Seo, 2015a: 306

##### Type species.

*Longileptoneta songniensis* Seo, 2015 from South Korea.

##### Diagnosis.

**Figure 5.**
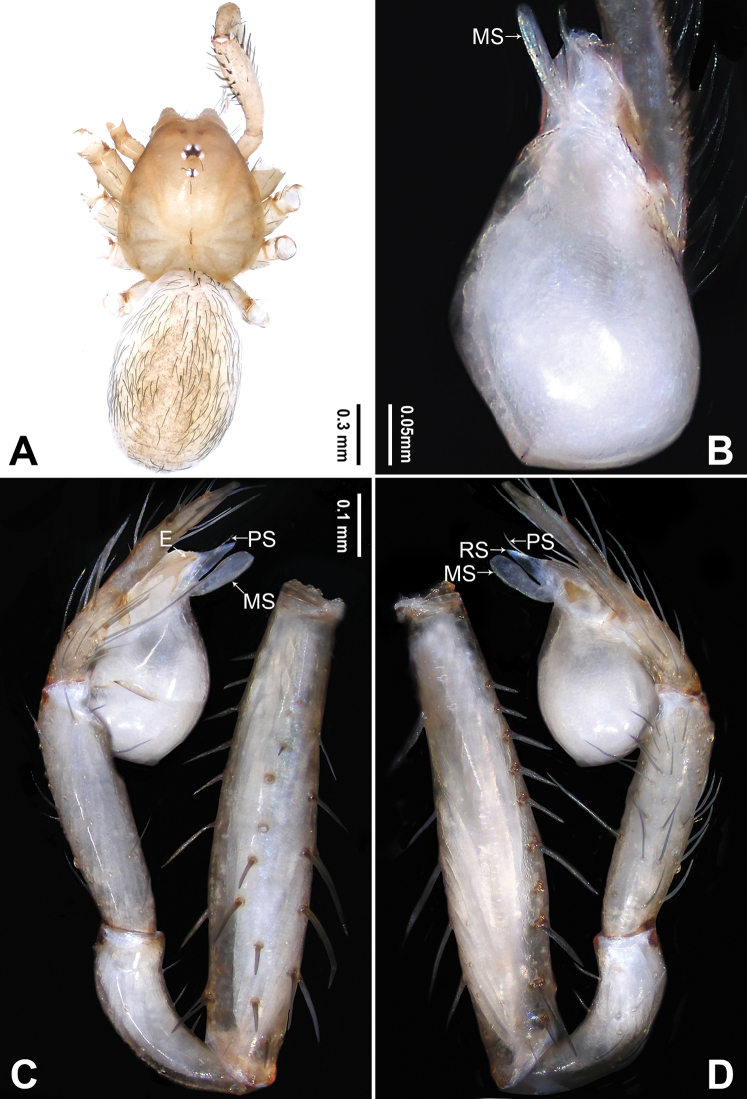
*Longileptoneta buyongsan* sp. nov., holotype male **A** habitus, dorsal view **B** palpal bulb, ventral view **C** palp, prolateral view **D** palp, retrolateral view. Abbreviations: **E** embolus **MS** median sclerite **PS** prolateral sclerite **RS** retrolateral sclerite. Scale bars: equal for **C, D**.

The genus *Longileptoneta* is similar to *Falcileptoneta* and *Leptoneta* in having few sclerites on the bulb and absenting cribellum but can be distinguished by the following combination of male palpal characters: femur with many strong spines (Figs 5C, D, 8A, B, 10C, D); tibia without apophyses or with simple apophyses (Figs 5D, 7D, 8B, 10D); tarsus usually with the prolateral curvature bearing prolaterodistal spurs (Figs 5C, D, 8A, B, 10C, D); bulb usually with leaf-like embolus, narrow and nearly ribbon-like prolateromesal sclerites, serrated tip, transparent and tongue-like retrolateral sclerite (Figs 5B–D, 7B–D, 10B–D).

#### Longileptoneta buyongsan sp. nov.

Taxon classificationAnimaliaAraneaeLeptonetidae

0ECA55E5-44DB-5CA8-A0B5-DC663AF9231B

http://zoobank.org/B2B9B136-76D5-49DA-B56E-11127DF24DA5

Figures 5 , 6 , 12

##### Type material.

***Holotype*.** Male (NIBR), South Korea, Chungcheongbuk-do, Eumseong-gun, Eumseong-eup, Sajeong-ri, Mt. Buyongsan (36.970236°N, 127.619596°E, elevation ca 167 m), 6 August 2019, ZG. Chen, Z. Zhao & YY. Hu leg. ***Paratypes*.** 1 male and 1 female (NIBR), same data as holotype.

##### Diagnosis.

*Longileptoneta buyongsan* sp. nov. is similar to *L. gayaensis* Seo, 2016 and *L. songniensis* Seo, 2015 but can be distinguished by the palpal tibia with one small retrolaterodistal apophysis (Fig. 5D) (vs. tibia without apophysis in *L. gayaensis* and *L. songniensis*); by the palpal tarsus without spur at tip (Fig. 5C, D) (vs. tarsus with one spur at tip in *L. gayaensis*; tarsus with one spur at tip and one prolateroproximal spur, branched retrolaterally in *L. songniensis*); also distinguished from *L. songniensis* by the palpal bulb with needle-shaped prolateral sclerite and shoehorn-like median sclerite (Fig. 5B–D) (vs. without prolateral sclerite and finger-like median sclerite in *L. songniensis*).

##### Description.

**Male** (holotype). Total length 1.86. Prosoma 0.82 long, 0.76 wide. Opisthosoma 1.04 long, 0.74 wide. Clypeus 0.11 high. Leg measurements: I 5.56 (1.60, 0.30, 1.67, 1.28, 0.71); II 4.50 (1.28, 0.26, 1.29, 1.03, 0.64); III 3.56 (1.03, 0.19, 0.89, 0.83, 0.62); IV 4.96 (1.41, 0.24, 1.47, 1.15, 0.69). Habitus as in Fig. 5A. Prosoma brownish. Eyes six (Fig. 5A). Median groove, cervical grooves and radial furrows distinct. Opisthosoma yellowish, ovoid. Palp (Fig. 5C, D): femur with many strong spines (Fig. 5C, D); tibia with one small retrolaterodistal apophysis (Fig. 5D); tarsus with many spines and prolateral curvature (Fig. 5C, D). Bulb with leaf-like embolus and three types of sclerites: prolateral sclerite needle-shaped; median sclerite shoehorn-like; retrolateral sclerite transparent and triangular (Fig. 5B–D).

**Figure 6.**
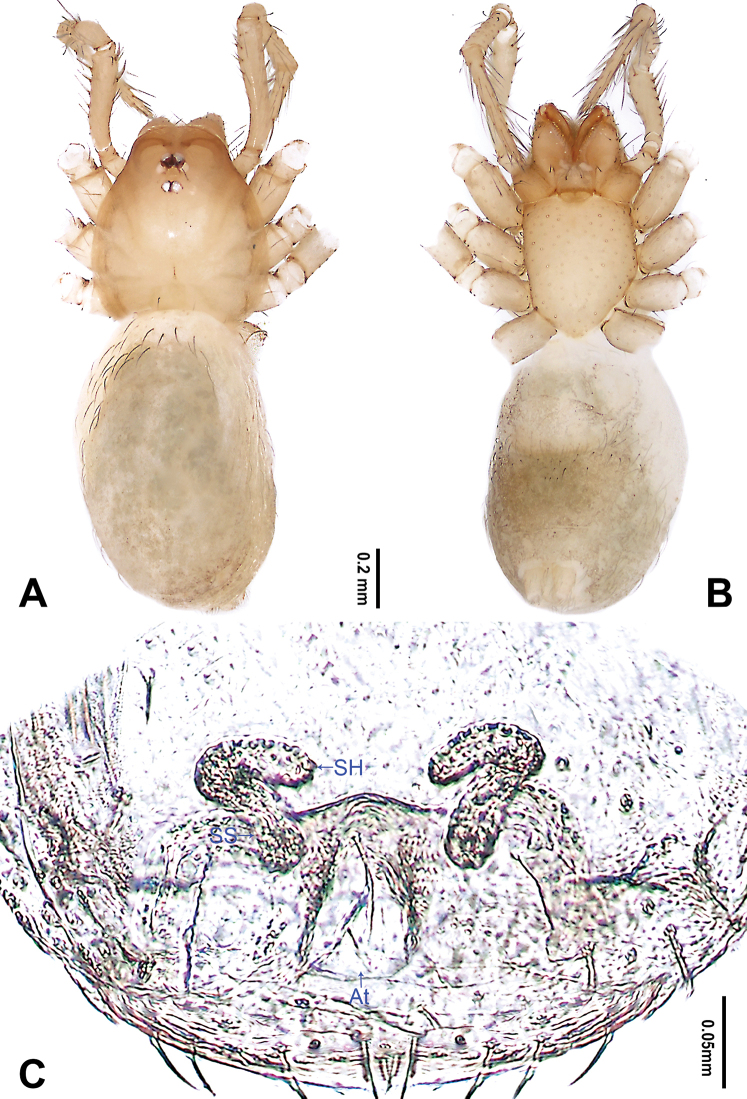
*Longileptoneta buyongsan* sp. nov., female paratype **A** habitus, dorsal view **B** habitus, ventral view **C** internal genitalia, dorsal view. Abbreviations: **At** atrium **SH** spermathecae **SS** spermathecae stalk. Scale bar: equal for **A, B**.

**Female** (one of the paratypes). Similar to male in color and general features, habitus as in Fig. 6A, B. Total length 1.77. Prosoma 0.71 long, 0.61 wide. Opisthosoma 1.06 long, 0.70 wide. Clypeus 0.10 high. Leg measurements: I 3.95 (1.09, 0.25, 1.14, 0.89, 0.58); II 3.13 (0.88, 0.20, 0.87, 0.63, 0.55); III – (0.71, -, -, -, -); IV 3.79 (1.08, 0.24, 1.07, 0.83, 0.57). Internal genitalia (Fig. 6C) with atrium rectangular, spermatheca and genital duct tube-shaped, loosely coiled.

##### Etymology.

The specific name refers to the type locality and is a noun in apposition.

##### Habitat.

Litter layers in mixed forest.

##### Distribution.

South Korea (Chungcheongbuk-do; Fig. 12).

#### Longileptoneta byeonsanbando sp. nov.

Taxon classificationAnimaliaAraneaeLeptonetidae

CCCB2A0C-F5F8-5AAA-A3BD-A3AF14D72987

http://zoobank.org/77E95657-EE0D-40ED-A697-8CC89B6C4B7D

**Figure 7.**
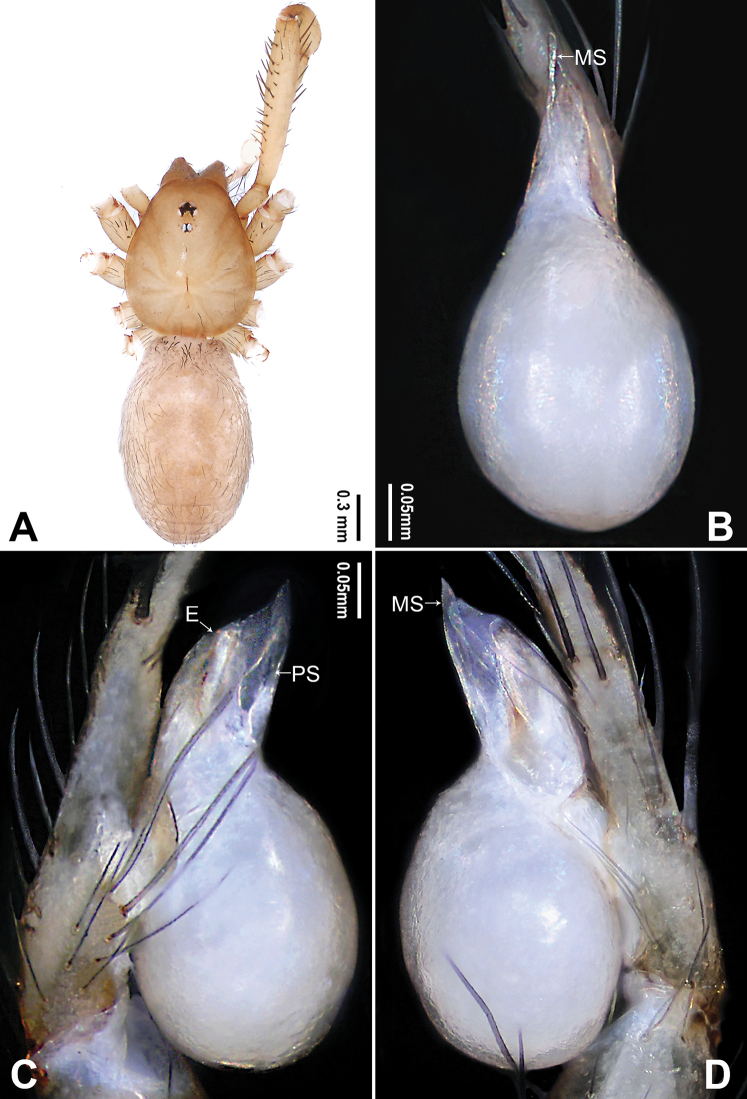
*Longileptoneta byeonsanbando* sp. nov., holotype male **A** habitus, dorsal view **B** palpal bulb, ventral view **C** palpal bulb, prolateral view **D** palpal bulb, retrolateral view. Abbreviations: **E** embolus **MS** median sclerite **PS** prolateral sclerite. Scale bars: equal for **C, D**.

**Figure 8.**
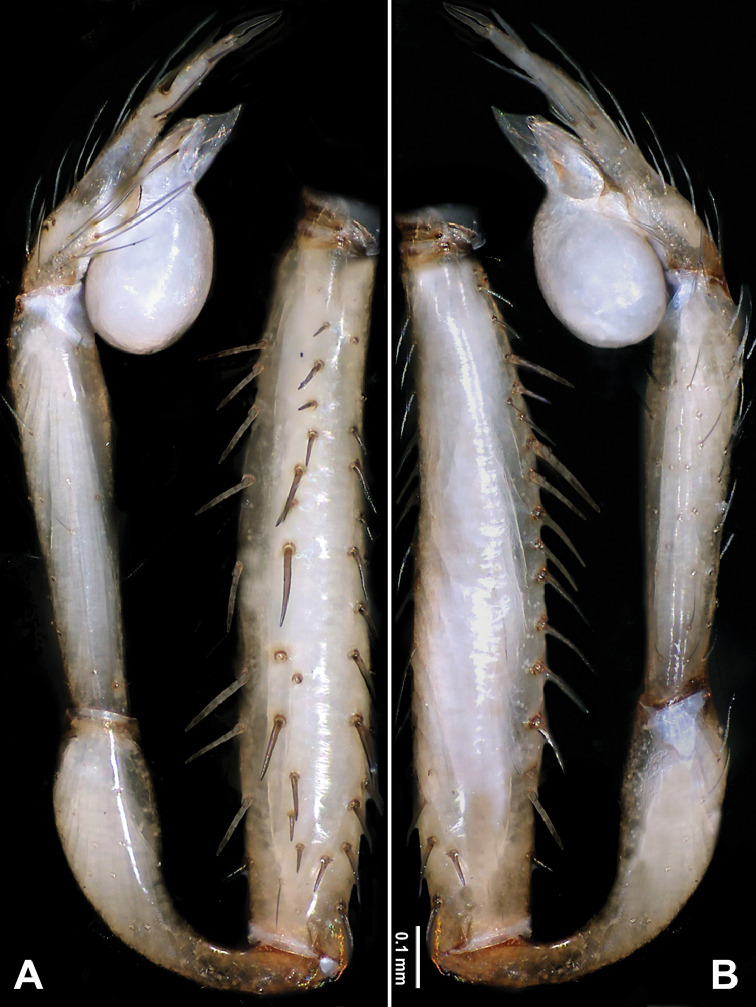
*Longileptoneta byeonsanbando* sp. nov., holotype male **A** palp, prolateral view **B** palp, retrolateral view. Scale bars: equal for **A, B**.

**Figure 9.**
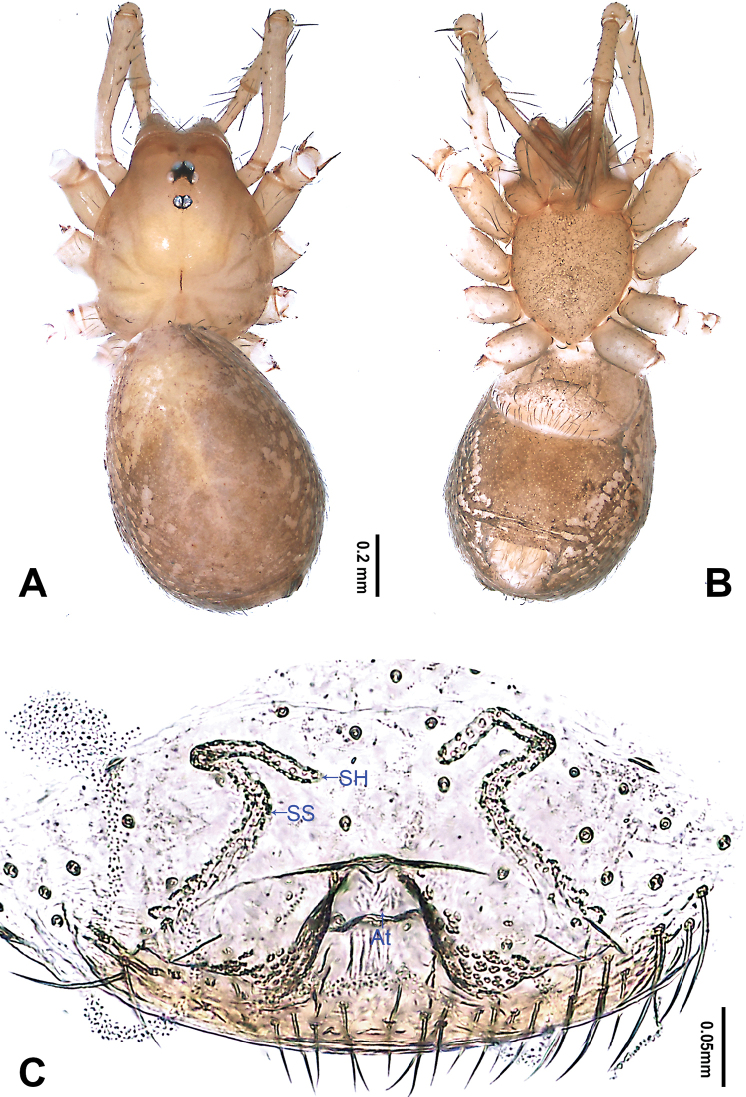
*Longileptoneta byeonsanbando* sp. nov., female paratype **A** habitus, dorsal view **B** habitus, ventral view **C** internal genitalia, dorsal view. Abbreviations: **At** atrium **SH** spermathecae **SS** spermathecae stalk. Scale bars: equal for **A, B**.

Figures 7 , 8 , 9 , 12

##### Type material.

***Holotype*.** Male (NIBR), South Korea, Jeollabuk-do, Buan-gun, Sangseo-myeon, Cheongrim-ri, Byeonsanbando National Park (35.670146°N, 126.629253°E, elevation ca 135 m), 8 August 2019, ZG. Chen, Z. Zhao & YY. Hu leg. ***Paratypes*.** 1 male and 1 female (NIBR), same data as holotype.

##### Diagnosis.

*Longileptoneta byeonsanbando* sp. nov. is similar to *L. gayaensis* Seo, 2016 and *L. jangseongensis* Seo, 2016 but can be distinguished by the palpal tibia with one distal columnar apophysis, with apophysis tip armed with one long spine retrolaterally (Figs 7D, 8B) (vs. tibia without apophysis in *L. gayaensis*; tibia with one small apophysis armed with one spine in *L. jangseongensis*); by the palpal bulb with narrow, leaf-like prolateral sclerite and ribbon-like median sclerite (Fig. 7B–D) (vs. needle-like prolateral sclerite and shoehorn-like median sclerite in *L. gayaensis*; without prolateral sclerite and leaf-like median sclerite in *L. jangseongensis*); and can be further distinguished from *L. gayaensis* by the presence of two spurs at tarsal tip (Fig. 8A, B) (vs. one spur in *L. gayaensis*).

##### Description.

**Male** (holotype). Total length 2.39. Prosoma 0.98 long, 0.86 wide. Opisthosoma 1.41 long, 0.90 wide. Clypeus 0.13 high. Leg measurements: I 6.81 (1.79, 0.33, 1.99, 1.67, 1.03); II 5.51 (1.56, 0.32, 1.54, 1.26, 0.83); III 4.43 (1.29, 0.26, 1.15, 1.02, 0.71); IV 6.15 (1.67, 0.32, 1.78, 1.47, 0.91). Habitus as in Fig. 7A. Prosoma brown. Eyes six (Fig. 7A). Median groove, cervical grooves and radial furrows distinct. Opisthosoma brown, ovoid. Palp (Figs 7C, D, 8A, B): femur with many strong spines and very long (Fig. 8A, B); patella very long (Fig. 8A, B); tibia with one distal columnar apophysis, with apophysis tip armed with one long spine retrolaterally (Figs 7D, 8B); tarsus with two spurs at tip and many spines, and with prolateral curvature (Fig. 8A, B). Bulb with leaf-like embolus and three types of sclerites: prolateral sclerite narrow, leaf-like; median sclerite ribbon-like; retrolateral sclerite with serrated tip, transparent and tongue-like (Fig. 7B–D).

**Female** (one of the paratypes). Similar to male in color and general features, habitus as in Fig. 9A, B. Total length 2.02. Prosoma 0.78 long, 0.68 wide. Opisthosoma 1.24 long, 0.86 wide. Clypeus 0.12 high. Leg measurements: I 5.23 (1.41, 0.31, 1.47, 1.14, 0.90); II 4.13 (1.16, 0.21, 1.15, 0.90, 0.71); III 3.33 (0.96, 0.19, 0.83, 0.77, 0.58); IV 4.61 (1.28, 0.32, 1.27, 1.03, 0.71). Internal genitalia (Fig. 9C): atrium trapezoidal, spermathecae and genital duct slender, tube-shaped, loosely coiled.

##### Etymology.

The specific name refers to the type locality and is a noun in apposition.

##### Habitat.

Litter layers in mixed forest.

##### Distribution.

South Korea (Jeollabuk-do; Fig. 12).

#### Longileptoneta jirisan sp. nov.

Taxon classificationAnimaliaAraneaeLeptonetidae

A3358BC3-81B1-5E51-AF3D-8345E5654A3B

http://zoobank.org/9BF96C1B-55D4-402C-8A33-1E0B9FAA1101

**Figure 10.**
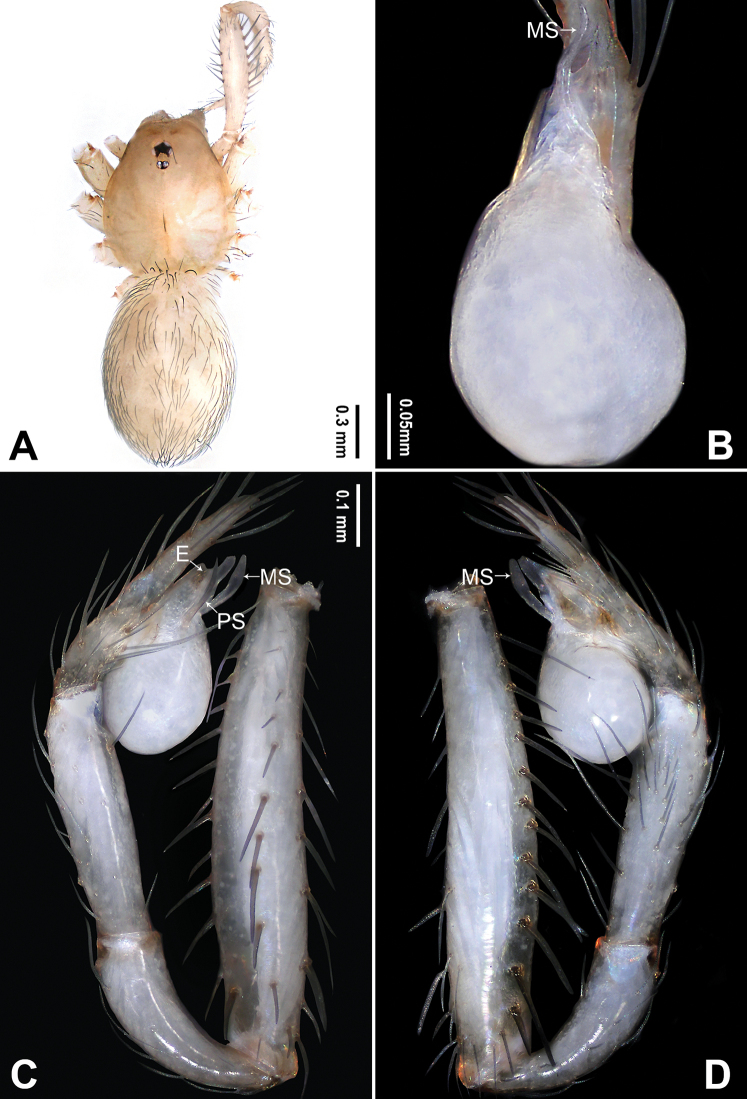
*Longileptoneta jirisan* sp. nov., holotype male **A** habitus, dorsal view **B** palpal bulb, ventral view **C** palp, prolateral view **D** palp, retrolateral view. Abbreviations: **E** embolus **MS** median sclerite **PS** prolateral sclerite. Scale bars: equal for **C, D**.

**Figure 11.**
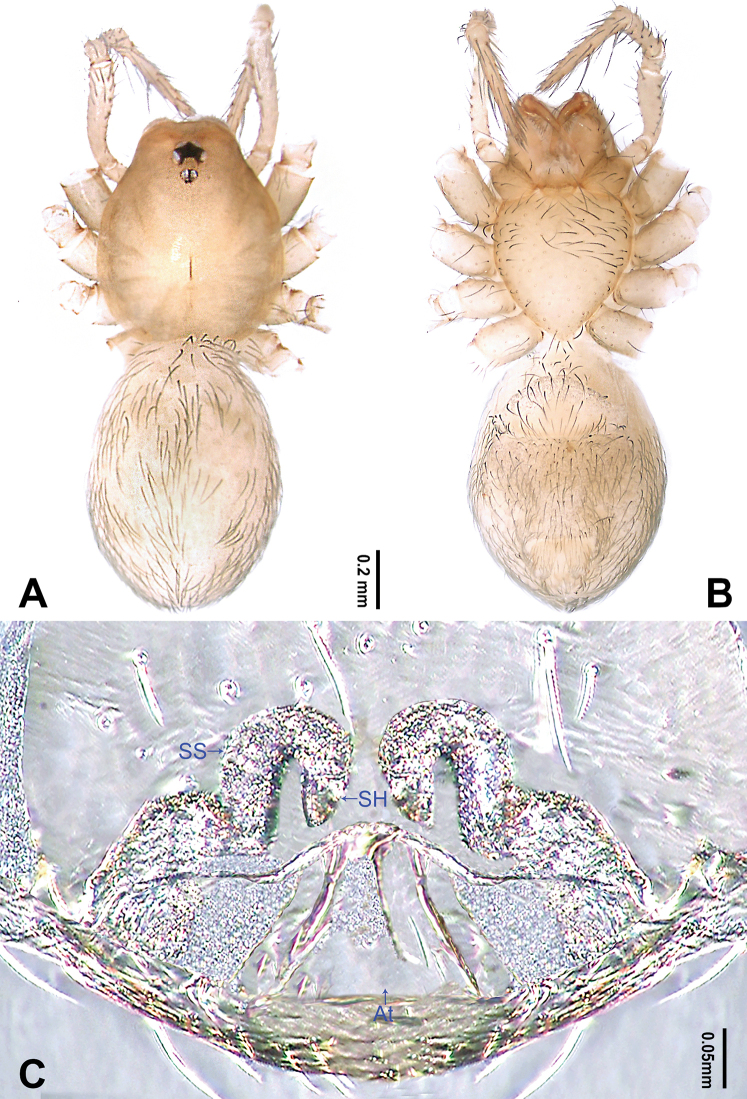
*Longileptoneta jirisan* sp. nov., female paratype **A** habitus, dorsal view **B** habitus, ventral view **C** internal genitalia, dorsal view. Abbreviations: **At** atrium **SH** spermathecae **SS** spermathecae stalk. Scale bars: equal for **A, B**.

**Figure 12.**
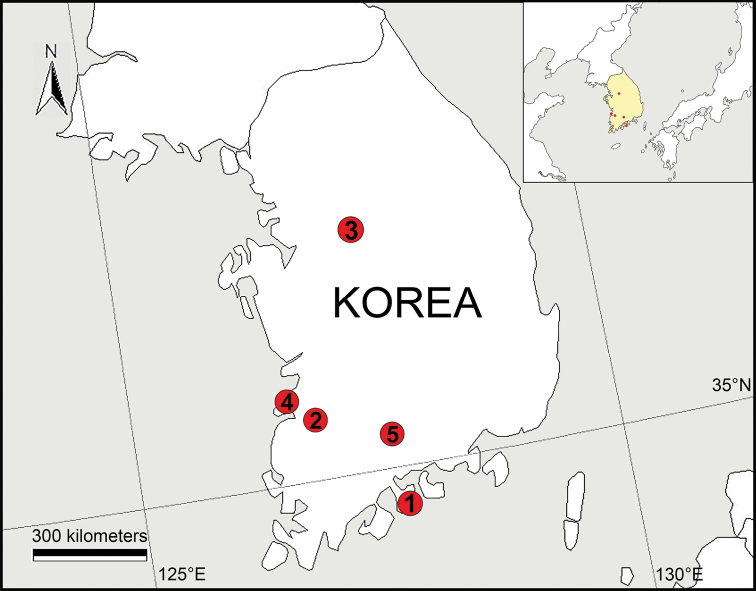
Known distribution records of new leptonetid species from Korea **1***Falcileptoneta dolsan* sp. nov. **2***Falcileptoneta naejangsan* sp. nov. **3***Longileptoneta buyongsan* sp. nov. **4***Longileptoneta byeonsanbando* sp. nov. **5***Longileptoneta jirisan* sp. nov.

Figures 10 , 11 , 12

##### Type material.

***Holotype*.** Male (NIBR), South Korea, Gyeongsangnam-do, Hadong-gun, Hwagye-myeon, Daeseong-ri, Mt. Jirisan National Park (35.273974°N, 127.657439°E, elevation ca 357 m), 15 August 2019, ZG. Chen, Z. Zhao & YY. Hu leg. ***Paratype*.** 1 female (NIBR), same data as holotype.

##### Diagnosis.

*Longileptoneta jirisan* sp. nov. is similar to *L. gachangensis* Seo, 2016 and *L. weolakensis* Seo, 2016 but can be distinguished by the palpal tibia with one retrolaterodistal spur (Fig. 10D) (vs. tibia without apophysis in *L. gachangensis* and *L. weolakensis*); and by the palpal bulb with spur-like prolateral sclerite and tongue-like retrolateral sclerite (Fig. 10C, D) (vs. blade-like prolateral sclerite and skinny and triangular retrolateral sclerite in *L. gachangensis*; without prolateral sclerite and triangular retrolateral sclerite in *L. weolakensis*).

##### Description.

**Male** (holotype). Total length 1.85. Prosoma 0.78 long, 0.72 wide. Opisthosoma 1.07 long, 0.76 wide. Clypeus 0.10 high. Leg measurements: I 5.52 (1.60, 0.27, 1.67, 1.35, 0.63); II 4.50 (1.35, 0.25, 1.28, 1.03, 0.59); III 3.68 (1.04, 0.19, 0.96, 0.91, 0.58); IV 5.04 (1.47, 0.26, 1.53, 1.16, 0.62). Habitus as in Fig. 10A. Prosoma brownish. Eyes six (Fig. 10A). Median groove, cervical grooves and radial furrows distinct. Opisthosoma yellowish, ovoid. Palp (Fig. 10C, D): femur with many strong spines (Fig. 10C, D); tibia with one retrolaterodistal spur (Fig. 10D); tarsus with two spurs at tip and many spines, and with prolateral curvature (Fig. 10C, D). Bulb with leaf-like embolus and three types of sclerites: prolateral sclerite spur-like; median sclerite leaf-like; retrolateral sclerite with serrated tip, transparent and tongue-like (Fig. 10B–D).

**Female** (paratype). Similar to male in color and general features, habitus as in Fig. 11A, B. Total length 1.76. Prosoma 0.74 long, 0.62 wide. Opisthosoma 1.02 long, 0.68 wide. Clypeus 0.10 high. Leg measurements: I 4.43 (1.28, 0.26, 1.34, 0.97, 0.58); II 3.44 (0.96, 0.22, 0.94, 0.76, 0.56); III 2.92 (0.83, 0.19, 0.74, 0.66, 0.50); IV 4.10 (1.17, 0.24, 1.16, 0.97, 0.56). Internal genitalia (Fig. 11C) with atrium trapezoidal, spermatheca and genital duct tube-shaped, loosely coiled.

##### Etymology.

The specific name refers to the type locality and is a noun in apposition.

##### Habitat.

Litter layers in mixed forest.

##### Distribution.

South Korea (Gyeongsangnam-do; Fig. 12).

## Supplementary Material

XML Treatment for Falcileptoneta

XML Treatment for Falcileptonetadolsan

XML Treatment for Falcileptoneta naejangsan

XML Treatment for Longileptoneta

XML Treatment for Longileptoneta buyongsan

XML Treatment for Longileptoneta byeonsanbando

XML Treatment for Longileptoneta jirisan
